# Comparison between functional lung volume measurement and segment counting for predicting postoperative pulmonary function after pulmonary resection in lung cancer patients

**DOI:** 10.1186/s12890-022-02299-y

**Published:** 2023-01-05

**Authors:** Zheyuan Fan, Shilei Zhao, Ling Wang, Fengzhou Li, Jin Wang, Chundong Gu

**Affiliations:** 1 Department of Cardiothoracic Surgery, The Affiliated Jinyang Hospital of Guizhou Medical University, Guiyang, 550023 China; 2 Dalian Medical University, Dalian, 116044 Liaoning China; 3 Department of Thoracic Surgery, The First Affiliated Hospital of Dalian Medical University, 222 Zhongshan Road, Dalian, 116011 Liaoning China; 4 Lung Cancer Diagnosis and Treatment Center of Dalian, The First Affiliated Hospital of Dalian Medical University, Dalian, 116011 China; 5 Department of Emergency Medicine, The First Affiliated Hospital of Dalian Medical University, Dalian, 116011 China

**Keywords:** Lung resection, Functional lung volume, Postoperative pulmonary function

## Abstract

**Background:**

Functional lung volume (FLV) obtained from computed tomography images was a breakthrough for lung imaging and functional assessment. We compared the accuracy of the FLV measurement method and the segment-counting (SC) method in predicting postoperative pulmonary function.

**Methods:**

A total of 113 patients who underwent two thoracoscopic surgeries were enrolled in our study. We predicted postoperative pulmonary function by the FLV measurement method and the SC method. Novel formulas based on the FLV measurement method were established using linear regression equations between the factors affecting pulmonary function and the measured values.

**Results:**

The predicted postoperative forced vital capacity (ppoFVC) and forced expiratory volume in 1 s (ppoFEV1) measured by the 2 methods showed high concordance between the actual postoperative forced vital capacity (postFVC) and the forced expiratory volume in 1 s (postFEV1) [*r* = 0.762, *P* < 0.001 (FLV method) and *r* = 0.759, *P* < 0.001 (SC method) for FVC; *r* = 0.790, *P* < 0.001 (FLV method) and *r* = 0.795, *P* < 0.001 (SC method) for FEV1]. Regression analysis showed that the measured preoperative pulmonary function parameters (FVC, FEV1) and the ratio of reduced FLV to preoperative FLV were significantly associated with the actual postoperative values and could predict these parameters (all *P* < 0.001). The feasibility of using these equations [postFVC = 0.8 × FVC − 0.784 × ΔFLV/FLV + 0.283 (R^2^ = 0.677, RSD = 0.338), postFEV1 = 0.766 × FEV1 − 0.694 × ΔFLV/FLV + 0.22 (R^2^ = 0.743, RSD = 0.265)] to predict the pulmonary function parameters after wedge resection was also verified.

**Conclusions:**

The new FLV measurement method is valuable for predicting postoperative pulmonary function in patients undergoing lung resection surgery, with accuracy and consistency similar to those of the conventional SC method.

## Introduction

Anatomic lobectomy and systematic lymph node dissection are the principal treatments for lung cancer. However, patients with poor pulmonary function or complications are not suitable for surgical resection. The prediction of postoperative pulmonary function is useful for identifying patients at increased risk for medical complications after lung cancer resection [1]. The guidelines recommend that the preoperative physiologic assessment begins with spirometry to measure the forced expiratory volume in 1 s (FEV1) and carbon monoxide diffusion capacity (DLCO). Predicted postoperative (ppo) lung functions should be calculated. When ppoFEV1 < 30% or ppoDLCO < 30%, the risk of perioperative death and cardiopulmonary complications after anatomic lobectomy are significantly increased, according to the American College of Chest Physicians [2].

Lobectomy redistributes the blood flow and ventilation of the lungs. Compensatory swelling of the residual lung, displacement of the mediastinum, lifting of the diaphragm, and collapse of the thorax all complicate the assessment of postoperative pulmonary function [3]. Current guidelines hold that postoperative pulmonary function is most commonly predicted by a simple calculation using the lung segment counting (SC) method [2, 4], which may be inaccurate for predictions of residual pulmonary function, as it is based solely on the number of remaining pulmonary segments without considering that the volume and function of each lung segment are different [5, 6]. There are interindividual differences or variations in the volume or function of each segment, and underlying lung diseases such as atelectasis, pulmonary emphysema, and fibrosis sometimes distribute heterogeneously [7, 8]. These issues are expected to interfere with the prediction of postoperative residual pulmonary function.

Thanks to great advances in computed tomography (CT) imaging technology, functional lung volume (FLV) measurement can successfully predict postoperative pulmonary function [9–13]. The ratio of the FLV to the total lung volume can be used to predict postoperative pulmonary function [5, 14]. Several studies have described a threshold point at which the lung is defined as functional [15]. Ueda K and coworkers defined the normal-attenuation areas (− 600 to − 910 HU), representing normal lung fields. They found that quantitative CT (QCT) more accurately predicted functional reserve after lung cancer surgery and identified patients whose lung functional assessment may be underestimated. Thus, FLV measurement is better for estimating the functional contribution of specific resected segments and quantifying the volume of the lung with normal structure [10].

In this retrospective study, we aimed to compare the FLV measurement method based on CT image analysis with the traditional SC method to predict several postoperative lung parameters in patients in a lobectomy cohort. Additionally, we developed improved formulas for predicting these postoperative lung parameters based on measured pulmonary function and pre- and post-pulmonary resection variables and validated the accuracy and precision of these equations.

## Material and methods

### Patients

This was a retrospective analysis of the clinical data of 236 patients who underwent two lung resection operations at the First Affiliated Hospital of Dalian Medical University from January 2011 to December 2018. The inclusion criteria were as follows: patients who underwent anatomical lung resection for lung cancer and who underwent pulmonary function tests, including thin-slice high-resolution CT examinations. The preoperative and postoperative pulmonary function tests and CT scans were performed within 1 week prior to surgery and at approximately 10 months after surgery for each patient in the seated position. The second preoperative examination included pulmonary function tests and chest CT examinations. Thus, the complete preoperative and postoperative pulmonary function values and CT images of each patient were obtained for further analysis. Patients were excluded if they underwent thoracotomy, thoracoscopic surgery for pneumothorax, or simultaneous bilateral lung resection surgery or had a past history of preoperative radiotherapy, tuberculosis or extensive pleural adhesions. Finally, 113 patients were enrolled: the first surgical procedure was video-assisted thoracic surgery (VATS) lobectomy in 56 patients and VATS sublobar resection in 57 patients (segmentectomy in 9 patients and wedge resection in 48 patients). The study was approved by the Medical Ethics Committee of the First Affiliated Hospital of Dalian Medical University (PJ-KS-KY-2021-127).

### Pulmonary function tests

Pulmonary function tests were performed by the same professional technicians at the First Affiliated Hospital of Dalian Medical University in the pulmonary function room. A 1085-series plethysmograph (Medical Graphic, USA) was used for the preoperative examination. The postoperative pulmonary function parameters referred to the same patient's second preoperative parameters. The pre- and postoperative measurement indicators included FVC and FEV1.

### CT examination

All CT images were obtained using a 64-row spiral CT system (SOMATOM Perspective, Siemens). Axial images were obtained from the lung apex to the lung base at full inspiration in the supine position. The following scanning parameters were used: tube voltage, 120 kV; tube current, 170 ~ 200 mAs; slice thickness, 5.00 mm; slice interval, 5.00 mm; matrix, 512 × 512; bone algorithm reconstruction, thin-slice thickness, 1.00 mm; slice interval, 1.00–1.25 mm; lung window width, 1000 Hounsfield units (HU), lung window level, − 600 HU; mediastinum window width, 400 HU; and mediastinum window level, 40 HU.

### CT image analyses

**Fig. 1:**
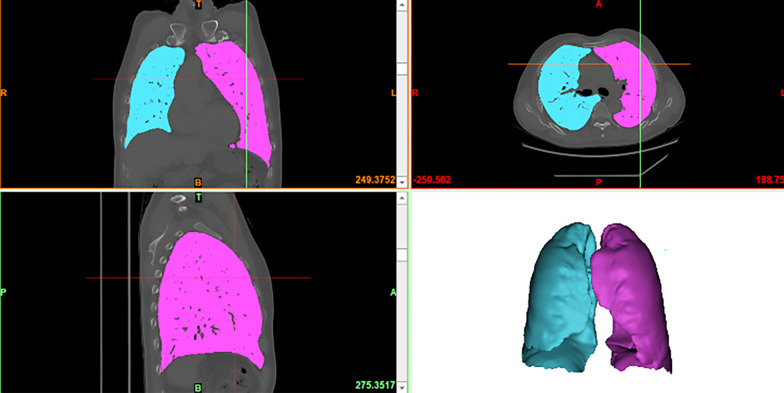
Coronal, axial and sagittal CT images and the TLV (CT threshold, − 1024 HU to − 500 HU)

All CT images of each patient were transferred to a workstation for visualizing and segmenting medical images and rendering three-dimensional (3D) objects (Mimics Medical 21.0 software, version 21.0, authorization number: 9E48-89C0-79F5-6F8D; Materialise, Belgium), on which 3D lung models were reconstructed. The 3D reconstruction model was extracted from the CT images using the algorithm of deep segmentation embedded in the software. The soft tissues and large blood vessels surrounding the lungs, atelectasis, fibrosis, and lung tumors could be excluded from the regions of interest after automatic tracheal reconstruction according to the preset CT threshold of − 500 HU to − 1024 HU. Boolean operations were used via the software to remove the volume occupied by the trachea, and the software automatically reconstructed and measured the total lung volume (TLV) (Fig. 1) [16, 17].

**Fig. 2:**
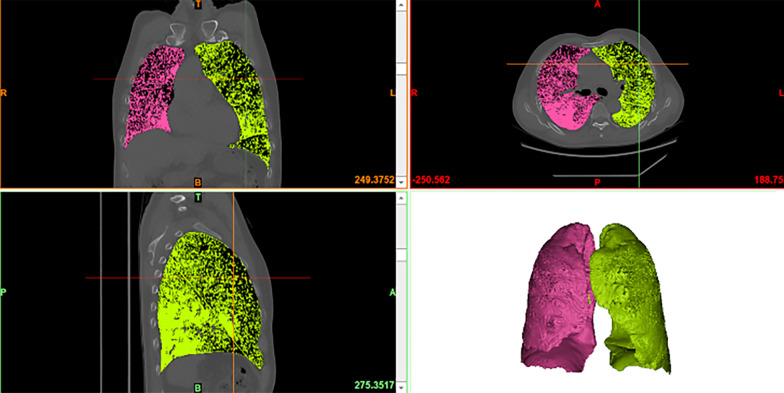
Coronal, axial and sagittal CT images and the FLV (CT threshold, − 910 HU to − 600 HU)

The CT threshold was adjusted from − 600 HU to − 910 HU using the same method as that applied to the TLV, and the lung parenchyma in the region with obstructed expiration was excluded. This radiologically defined lung volume is referred to as the FLV [5, 16]. The Mimics medical software automatically identified pulmonary fissures, and manual adjustments were applied to a few incorrectly identified fissures to distinguish different lobes and measure the FLV of each lobe, including the resected lobe volume (Fig. 2).

A board-certified surgeon specializing in lung imaging analyzed the CT images, and a single radiologist with expertise in chest radiography reviewed all the fused 3D reconstruction images. Both of them were blinded to the patient’s clinical status and pulmonary functional test results.

### Prediction of postoperative pulmonary function

The FLV measurement method emphasizes the contribution of the resected lung parenchyma to overall pulmonary function, and the prediction of postoperative pulmonary function was based on the following formula [16]:

Predicted postoperative FVC_FLV_, FEV1_FLV_ (ppoFVC_FLV_, ppoFEV1_FLV_) = preoperative FVC, FEV1 × (1 − resected FLV/total FLV).

The SC method used to predict postoperative pulmonary function is based on the following formula:

Predicted postoperative FVC_SC_, FEV1_SC_ (ppoFVC_SC_, ppoFEV1_SC_) = preoperative FVC, FEV1 × (1 − number of resected pulmonary segments/19).

The total number of lung segments was 19, including 10 in the right lung (3 in the upper lobe, 2 in the middle lobe, and 5 in the lower lobe) and 9 in the left lung (5 in the upper lobe and 4 in the lower lobe). Each lung segment had the same volume, accounting for 5.26% of the total volume [2, 5, 18].

### Statistical analysis

Statistical analysis was performed using SPSS v22.0 (IBM, NY, USA). The two-sample independent t test was used to determine the differences between the preoperative and postoperative pulmonary function values. The differences between postoperative pulmonary function and postoperative pulmonary function predicted by the FLV measurement method and the SC method were compared by one-way analysis of variance (ANOVA). Pearson’s correlation coefficient was calculated between the predicted and measured values of the FLV measurement method and of the SC method. Bland‒Altman analysis was used to evaluate the consistency of the predicted values with the actual postoperative pulmonary function values. A multiple linear regression model was built to correct the effects of confounders on the prediction of postoperative pulmonary function, and regression equations between the factors affecting pulmonary function and the measured values were established. Differences were considered statistically significant when *P* < 0.05.

## Results

### Baseline characteristics

The patients’ clinical characteristics are shown in Table 1. In this study, we enrolled a total of 113 patients who underwent anatomical lung resection for lung cancer. The evaluations were performed within 1 month prior to surgery. The postoperative pulmonary function and CT images refer to the second preoperative parameters of the same patient. The preoperative and postoperative pulmonary function values are shown in Table 1. No significant differences in the demographic characteristics or preoperative pulmonary function were noted between the two groups. Although no differences were detected in the comparison of postoperative pulmonary function between the two groups, significant differences were found when comparing the loss of pulmonary function after VATS. The FEV1 loss ratio (*P* = 0.022) after lobectomy was significantly higher than that after sublobar resection.

**Table 1:** Clinical characteristics

Variables	Lobectomy (*n* = 56)	Sublobar resection (*n* = 57)	*P* value
Age, years	62.32 ± 7.72	60.26 ± 9.54	0.210
Male, n (%)	24 (42.9)	15 (26.3)	0.064
Smoking history, n (%)	10 (17.9)	6 (10.5)	0.264
Resected lobe			
Right upper lobe	16	11	
Right middle lobe	5	3	
Right lower lobe	6	8	
Left upper lobe	18	15	
Left lower lobe	9	6	
Others	2	14	
Interval between two operations (month)	10.51 (4.17–24.73)	3.57 (1.62–8.92)	0.004
Preoperative			
preFVC (L)	2.92 ± 0.59	3.04 ± 0.77	0.367
preFEV1 (L)	2.41 ± 0.56	2.48 ± 0.67	0.498
Postoperative			
postFVC (L)	2.58 ± 0.58	2.76 ± 0.71	0.141
postFEV1 (L)	2.05 ± 0.51	2.24 ± 0.61	0.065
FVC loss (%)	11.39 ± 13.52	8.16 ± 13.33	0.203
FEV1 loss (%)	14.37 ± 13.66	8.65 ± 12.58	0.022

FEV1, forced expiratory volume in 1 s; FVC, forced vital capacity; postFVC, postoperative forced vital capacity; postFEV1, postoperative forced expiratory volume in 1 s; preFVC, preoperative forced vital capacity; preFEV1, preoperative forced expiratory volume in 1 s

**Fig. 3:**
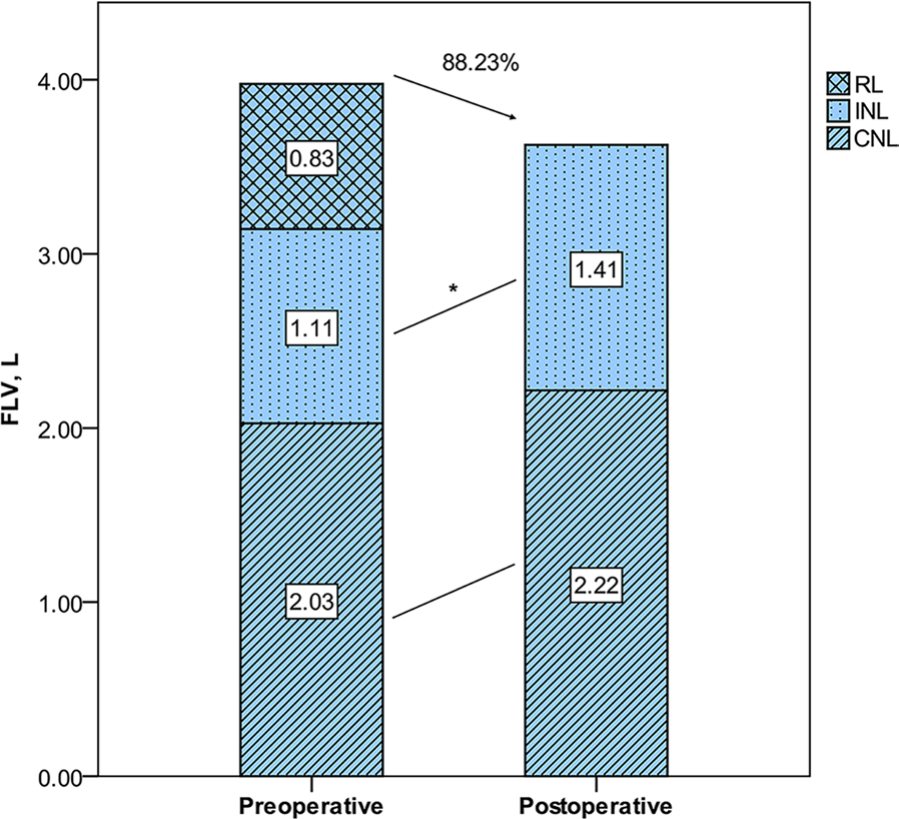
FLV before and after thoracoscopic lobectomy. The volume of each part is expressed as the mean in our cohort. FLV, functional lung volume; RL, resected lobe; INL, ipsilateral nonoperated lobe; CNL, contralateral nonoperated lobe. *P < 0.05 versus preoperative ipsilateral nonoperated lobe

The proportional FLV of each lobe according to the FLV measurement method is shown in Table 2. Figure 3 shows the change in the FLV following lobectomy. After thoracoscopic lobectomy, the postoperative FLV of the unaffected ipsilateral lobe was significantly greater than the preoperative value (*P* < 0.05). In contrast, the postoperative FLV of the contralateral nonoperated lobe tended to be similar to the preoperative value. The correlations between the measured volumetric values and the preoperative and postoperative pulmonary function values in the lobectomy group are shown in Table 3.

**Table 2:** Proportional FLV of each lobe according to the FLV measurement method

Variables	FLV (mL)	Proportion of ipsilateral FLV (%)	Proportion of total FLV (%)
RL	2240.44 ± 579.82	100.00	54.21
LL	1895.54 ± 499.92	100.00	45.79
RUL	844.91 ± 277.99	37.62	20.39
RML	396.12 ± 116.11	18.00	9.74
RLL	982.97 ± 256.42	52.24	23.91
LUL	999.42 ± 300.19	44.38	24.08
LLL	912.56 ± 281.62	47.76	21.88

RL, right lobe; LL, left lobe; RUL, right upper lobe; RML, right middle lobe; RLL, right lower lobe; LUL, left upper lobe; LLL, left lower lobe

**Table 3:** Correlation between lung volume measured by the volumetric method and preoperative and postoperative pulmonary function

Variable	FVC		FEV1	
	*r*	*P*	*r*	*P*
FLV				
Preoperative	0.610	0.000	0.421	0.001
Postoperative	0.634	0.000	0.508	0.000
TLV				
Preoperative	0.684	0.000	0.473	0.000
Postoperative	0.557	0.000	0.425	0.001

FEV1, forced expiratory volume in 1 s; FLV, functional lung volume; FVC, forced vital capacity; TLV, total lung volume

### Correlation and consistency of the FLV measurement method and the SC method

**Fig. 4:**
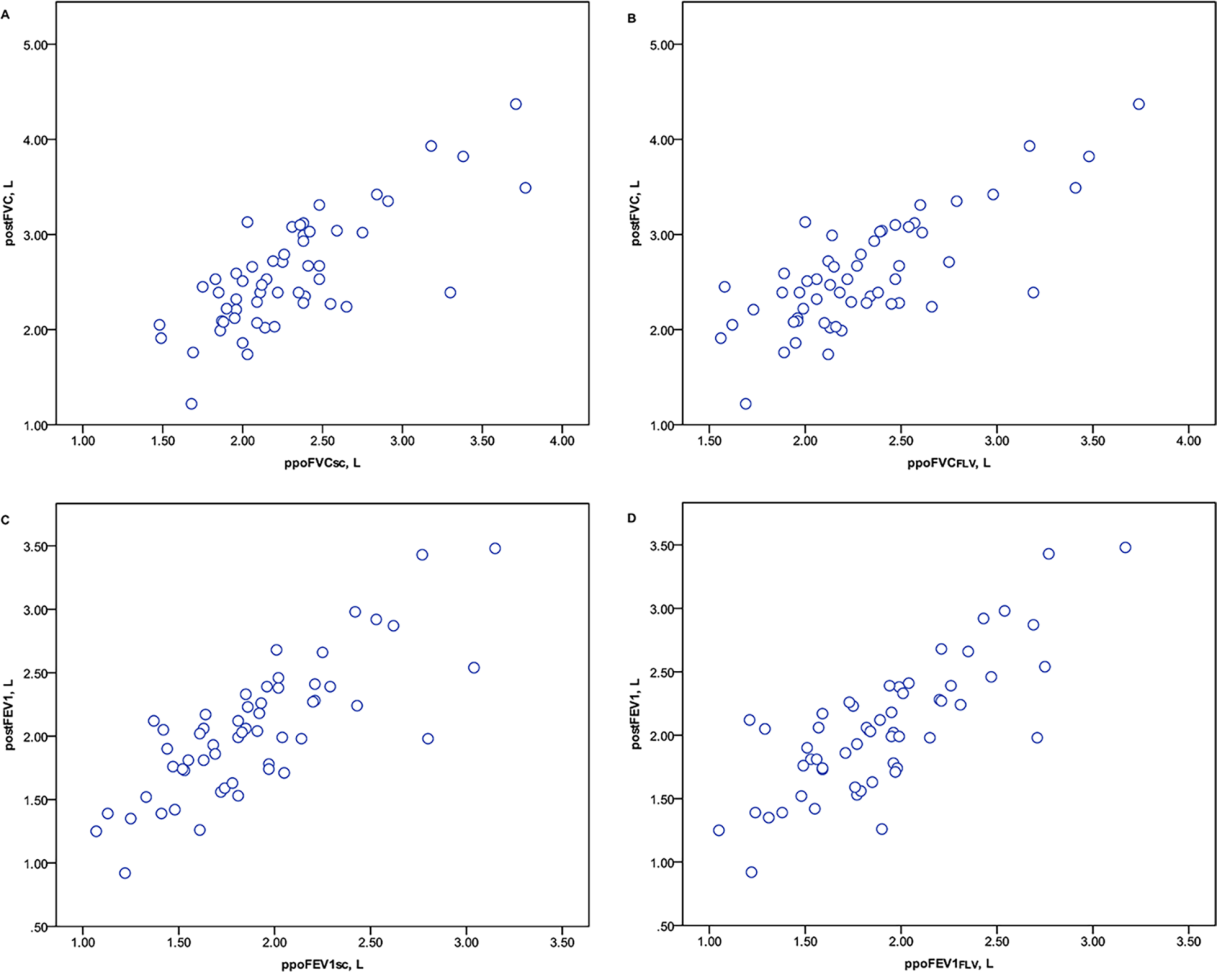
Relationship between postoperative pulmonary function and predicted values as determined by Pearson’s rank correlation coefficients (left, SC method; right, FLV measurement method). ppo, predicted postoperative; post, postoperative; FLV, functional lung volume; SC, segment counting; FVC, forced vital capacity; FEV1, forced expiratory volume in 1 s

**Fig. 5:**
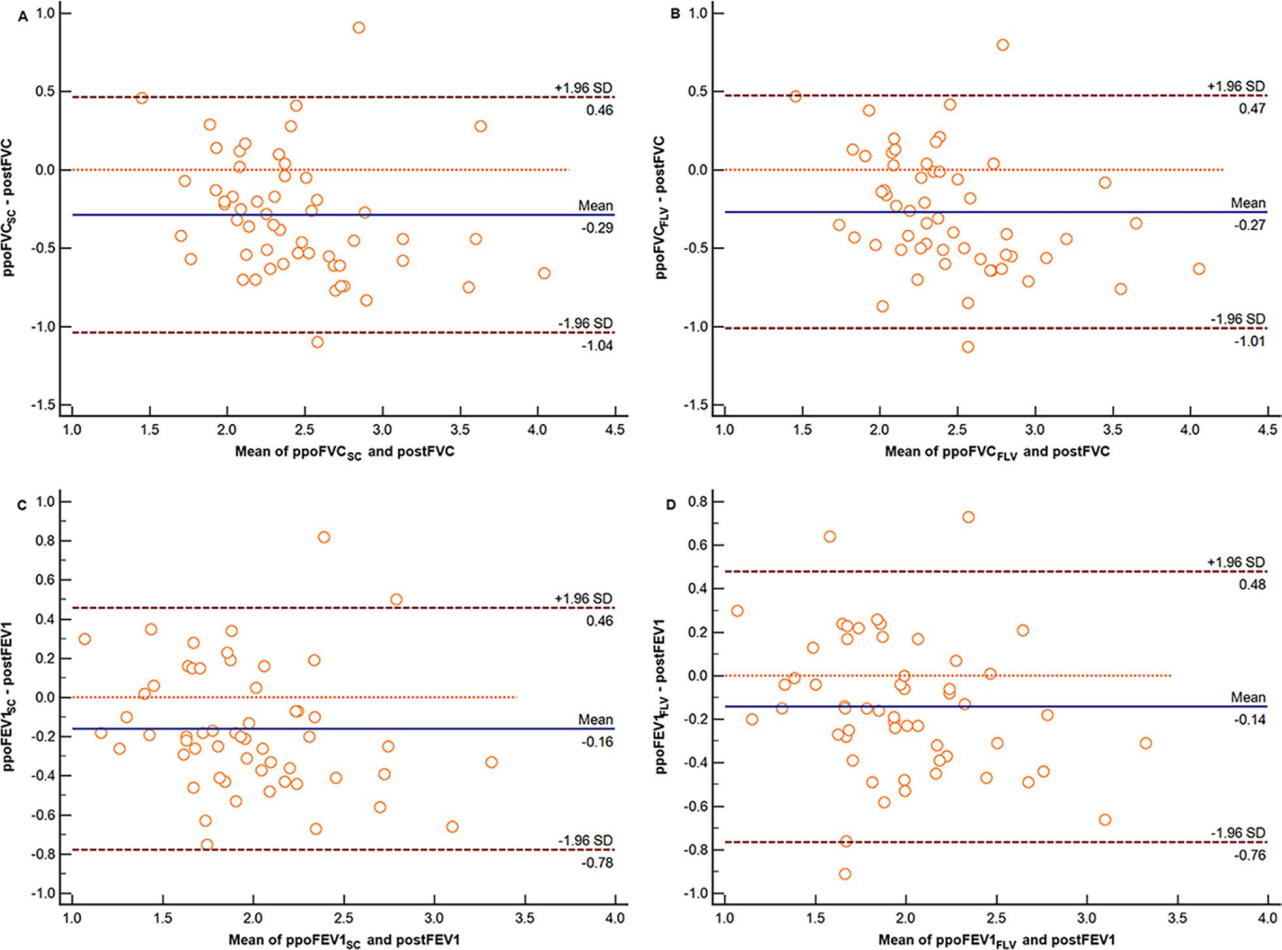
Bland‒Altman plots showing the mean difference (solid line) between the measured and predicted postoperative pulmonary function values with the limits of agreement (± 1.96 standard deviation, thick dotted lines) using the SC method (left) and the FLV measurement method (right). ppo, predicted postoperative; post, postoperative; FLV, functional lung volume; SC, segment counting; FVC, forced vital capacity; FEV1, forced expiratory volume in 1 s

In the lobectomy cohort, the relationships between the predicted and measured postoperative values (Table 4) and the reliability of the two methods of predicting postoperative pulmonary function are shown in Fig. 4. For all pulmonary function parameters, the correlation coefficients of the FLV measurement method were similar to the correlation coefficients for the SC method. The intraclass correlation coefficients showed similar tendencies. Given that the intraclass correlation coefficients were greater than 0.70, these two methods showed excellent reliability in predicting the postoperative FVC and FEV1 (Table 5). The agreement between the measured and predicted values obtained using the two methods is shown in Fig. 5. The FLV measurement method showed better agreement with postoperative pulmonary function than the SC method for FVC [limits of agreement: − 1.01 to 0.47 (mean, − 0.27) vs. − 1.04 to 0.46 (mean, − 0.29)] and FEV1 [(− 0.76 to 0.48 (mean, − 0.14) vs. − 0.78 to 0.46 (mean, − 0.16)].

**Table 4:** Comparison of the measured and predicted postoperative pulmonary function values obtained using the FLV and SC methods

Variable	Postoperative pulmonary function	SC	FLV
FVC (L)	2.58 ± 0.59	2.29 ± 0.49*	2.31 ± 0.46*
FEV1 (L)	2.05 ± 0.51	1.89 ± 0.46	1.90 ± 0.44

FEV1, forced expiratory volume in 1 s; FLV, functional lung volume; FVC, forced vital capacity; SC, segment-counting

*P < 0.05 versus postoperative pulmonary function

**Table 5:** Correlation coefficients of the measured and predicted postoperative pulmonary function values obtained using the FLV and SC methods

Variables	Coefficient	*P* value
ppoFVC_SC_ × postFVC	0.759	< 0.001
ppoFVC_FLV_ × postFVC	0.762	< 0.001
ppoFEV1_SC_ × postFEV1	0.795	< 0.001
ppoFEV1_FLV_ × postFEV1	0.790	< 0.001

FEV1, forced expiratory volume in 1 s; FLV, functional lung volume; FVC, forced vital capacity; ppo, predicted postoperative; post, postoperative; SC, segment-counting

### Establishment and validation of novel equations for the prediction of postoperative pulmonary function

**Fig. 6:**
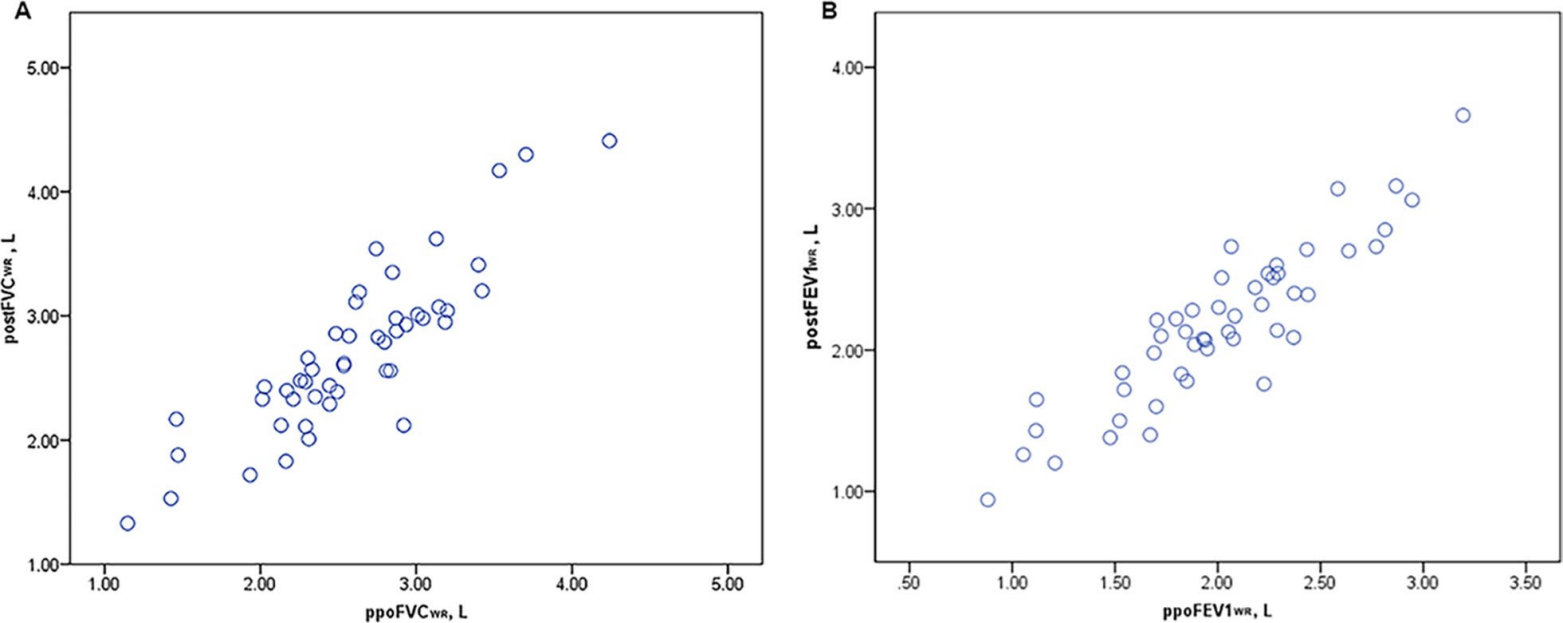
Relationship between the measured and predicted postoperative pulmonary function values following wedge resection as determined by Pearson’s rank correlation coefficient (left, FVC; right, FEV1). ppo, predicted postoperative; post, postoperative; FLV, functional lung volume; FVC, forced vital capacity; FEV1, forced expiratory volume in 1 s; WR, wedge resection

**Fig. 7:**
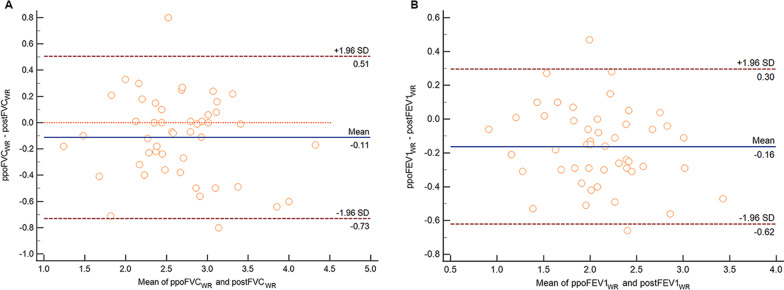
Bland‒Altman plots showing the mean difference (solid line) between the measured and predicted postoperative pulmonary function values following wedge resection using the FLV measurement method and the limits of agreement (± 1.96 standard deviation, thick dotted lines; left, FVC; right, FEV1). ppo, predicted postoperative; post, postoperative; FLV, functional lung volume; FVC, forced vital capacity; FEV1, forced expiratory volume in 1 s; WR, wedge resection

Table 6 shows the parameters related to postoperative pulmonary function based on multivariate and stepwise regression and the output of each variable included in the final linear regression model. The regression equations developed to calculate postoperative pulmonary function are presented in Table 7. The regression equations for the prediction of postoperative pulmonary function were verified in the wedge resection cohort (Figs. 6, 7). The Pearson correlation coefficients, intraclass correlation coefficients, and limits of agreement in this cohort were similar to those obtained in the lobectomy cohort, confirming the validity of these equations in the wedge resection cohort.

**Table 6:** Parameters related to postFVC and postFEV1 based on the multivariate and stepwise analyses

Variables	Multivariate analysis				Univariate analysis			
	Coefficient	*P* value	95% CI		Coefficient	*P* value	95% CI	
			Lower limit	Upper limit			Lower limit	Upper limit
PostFVC								
Constant	2.193	0.211	− 1.285	5.672	0.283	0.223	− 0.178	0.745
Sex	0.155	0.267	− 0.123	0.434				
Age	− 0.003	0.655	− 0.018	0.011				
Height	− 0.015	0.254	− 0.040	0.011				
Weight	0.009	0.080	− 0.001	0.018				
RL	− 1.864E−07	0.458						
INL	1.288E−07	0.388						
CNL	1.551E−07	0.290						
ΔFLV/FLV	− 0.978	0.001	− 1.506	− 0.449	− 0.784	0.001	− 1.225	− 0.344
FVC	0.722	0.000	0.446	0.998	0.800	0.000	0.645	0.956
PostFEV1								
Constant	0.250	0.859	− 2.569	3.070	0.220	0.173	− 0.099	0.539
Sex	0.098	0.381	− 0.125	0.321				
Age	− 0.004	0.474	− 0.016	0.007				
Height	0.001	0.922	− 0.019	0.021				
Weight	0.001	0.759	− 0.007	0.009				
RL	− 1.129E−07	0.562						
INL	4.045E−08	0.734						
CNL	1.015E−07	0.394						
ΔFLV/FLV	− 0.810	0.000	− 1.240	− 0.381	− 0.694	0.000	− 1.037	− 0.351
FEV1	0.690	0.000	0.503	0.876	0.776	0.000	0.646	0.906

CNL, contralateral nonoperated lobe; FEV1, forced expiratory volume in 1 s; FLV, functional lung volume; FVC, forced vital capacity; INL, ipsilateral nonoperated lobe; post, postoperative; RL, resected lobe

**Table 7:** Linear regression equations used to determine the postoperative pulmonary function parameters

Parameters	Predictive equation	R^2^	RSD
postFVC	0.8 × FVC-0.784 × ΔFLV/FLV + 0.283	0.677	0.33859
postFEV1	0.766 × FEV1-0.694 × ΔFLV/FLV + 0.22	0.743	0.26510

FEV1, forced expiratory volume in 1 s; FLV, functional lung volume; FVC, forced vital capacity; Post, postoperative

## Discussion

We compared the predictability of postoperative pulmonary function using the SC method and FLV measurement method. Our results show that the values of the postoperative pulmonary function parameters predicted by the FLV measurement method and the SC method closely reflected the measured pulmonary function in patients after lung resection surgery. No significant differences were noted between the two methods. However, compared with the SC method, the difference between the predicted and measured values was smaller by the FLV measurement method. Moreover, we developed a set of linear regression equations to predict postoperative pulmonary function parameters using the FLV measurement method, and these regression equations for the prediction of postoperative pulmonary function were verified in the wedge resection cohort. Wedge resection better preserves pulmonary function, with postoperative pulmonary function changes similar to those after thoracoscopic mediastinal surgery and postoperative lung ventilation/blood flow changes smaller than those after lobectomy. The indicators used in the FLV measurement method may be better for predicting postoperative pulmonary function in such patients.

Various methods, such as the conventional SC method, QCT, PS and single-photon-emission computed tomography/computed tomography (SPECT/CT), have been used to investigate postoperative pulmonary function. Ueda et al. compared the SC method and QCT to determine ppoFEV1 and reported that the two methods were almost equally accurate [10]. Arnon-Sheleg et al. compared SC, PS and SPECT/CT in the prediction of FEV1 and DLCO and found that the values predicted by these methods matched the actual postoperative FEV1 and DLCO values equally well [18]. Fernández-Rodríguez et al. reported that volumetric CT is an accurate method for predicting postoperative pulmonary function, with better accuracy than conventional SC and PS [19]. Among these methods, QCT imaging is fast, technically simple and performed by analyzing available data from preoperative chest CT scans, which are available in all cases given that preoperative chest CT is routinely performed in all cases of lung cancer.

Areas of the lung affected by diseases, such as atelectasis, pulmonary tuberculosis, bronchiectasis and emphysema, were excluded from the analysis before we set the attenuation ranges (− 910 HU to − 600 HU) [5, 9, 10, 16]. In the process of selecting the lung tissue according to the CT threshold using Mimics software, we found that most low-attenuation areas were located in the anterior and upper lung fields when patients were in the supine position. Ueda et al. reported that according to preoperative estimates, the FLV decreased by 16.5 ± 4.3% when removing the upper lobe and 25.9 ± 4.3% when removing the lower lobe (*P* < 0.001) [20]. This finding confirms that low-attenuation areas are mostly located in the anterior and upper lung fields [11]. The resection of lung tissue in emphysematous areas may result in the recovery of overall pulmonary function [16, 21]. However, there is some disagreement regarding the method used to count the number of resected left lung segments (or subsegments). Regarding 3D-CT, Kobayashi et al. emphasized that the proportion of segments in the left upper lobe was significantly larger than that in the left lower lobe (26% vs. 17%) [5]. Nevertheless, some scholars input 10 segments for each of the upper and lower lobes of the left lung in the SC method, which may underestimate the difference between the upper and lower lobes of the left lung [22].

The space left by lobectomy is filled by compensatory dilation of the residual lung, displacement of the mediastinum, lifting of the diaphragm, and collapse of the thoracic cage. The main driving force is compensated expansion of the ipsilateral nonoperated lobe [3, 12]. In this study, we also observed that ipsilateral nonoperated lobe dilation was more aggressive than contralateral lobe dilation. The extent of compensatory lung growth after lung resection is not completely understood. After lobectomy, the FVC loss was 11.39 ± 13.52%, and the FEV1 loss was 14.37 ± 13.66%. Previous studies have shown a 19.19% FVC loss and a 21.02% FEV1 loss at 6 months after surgery [9]. Additionally, comparing the measured values of postoperative pulmonary function with the values predicted by the SC method and the FLV measurement method, the predicted values were lower. Considering that the interval between the two operations in the patients in the lobectomy group was greater than 1 year on average, the difference may be attributable to the passage of time, which allowed further pulmonary function restoration [8, 23]. The data suggest that the FLV measurement method to predict postoperative FEV1 should be implemented when pulmonary resection is being considered in the treatment of high-risk surgical candidates.

### Limitations

Our proposed technique has advantages in predicting postoperative pulmonary function following both lobectomy and wedge resection, whereas the conventional SC method cannot be used to evaluate sublobar resection-based pulmonary function. This retrospective study does have some limitations. First, we only used FVC and FEV1 to predict postoperative pulmonary function. Although the guidelines recommend the evaluation of DLCO and the maximum rate of oxygen consumption (VO_2_max), these parameters were not routinely examined in our hospital. Second, we analyzed the data of patients who underwent two lung resection surgeries. The interval between the two surgeries significantly varied, and postoperative chest CT imaging and pulmonary function evaluations were not performed at the same predefined time point or interval. Third, the CT imaging data we evaluated were obtained during the end-inspiratory phase. According to the degree of inspiration achieved by the patient, the degree of chest fluctuation varied, and the corresponding measured FLV slightly differed. Finally, the current sample may not be large enough to perform high-quality analysis, and given the heterogeneity of the sample, we could not be certain which methods of predicting postoperative pulmonary function have any selective advantage. Hence, more patients need to be enrolled from multiple centers to validate these results.

## Conclusion

The postoperative pulmonary function (ppoFVC and ppoFEV1) values predicted by the FLV method reflect the measured values of pulmonary function (postFVC and postFEV1) and fit the actual physical variation better than the values predicted by the SC method. The improved formulas based on the FLV measurement method further accurately predict pulmonary function after surgery and provide a reference for postoperative pulmonary function evaluations.

## Data Availability

The data used to support the findings of this study are available from the corresponding author upon request.

## Abbreviations

CNL: Contralateral nonoperated lobe
FLV: Functional lung volume
INL: Ipsilateral nonoperated lobe
ppo: Predicted postoperative
QCT: Quantitative computed tomography
RL: Resected lobe
SC: Segment-counting
TLV: Total lung volume
FEV1: Forced expiratory volume in 1 s
MVV: Maximum voluntary ventilation
DLCO: Carbon monoxide diffusion capacity
FVC: Forced vital capacity
CT: Computed tomography
PS: Perfusion scintigraphy
VATS: Video-assisted thoracic surgery
